# Epigenome-wide analysis of T-cell large granular lymphocytic leukemia identifies BCL11B as a potential biomarker

**DOI:** 10.1186/s13148-022-01362-z

**Published:** 2022-11-14

**Authors:** Patricia Johansson, Teresa Laguna, Julio Ossowski, Vera Pancaldi, Martina Brauser, Ulrich Dührsen, Lara Keuneke, Ana Queiros, Julia Richter, José I. Martín-Subero, Reiner Siebert, Brigitte Schlegelberger, Ralf Küppers, Jan Dürig, Eva M. Murga Penas, Enrique Carillo-de Santa Pau, Anke K. Bergmann

**Affiliations:** 1 Faculty of Medicine, Institute of Cell Biology (Cancer Research), University of Duisburg-Essen, Virchowstr. 177, 45122 Essen, Germany; 2 Computational Biology Group, Precision Nutrition and Cancer Research Program, IMDEA Food Institute, 28049 Madrid, Spain; 3 Institute for Human Genetics, Christian-Albrechts-University Kiel and University Hospital Schleswig Holstein, Campus Kiel, Kiel, Germany; 4 Institute of Human Genetics, Medical School Hannover (MHH), Hannover, Germany; 5 Centre de Recherches en Cancérologie de Toulouse (CRCT), Université de Toulouse, CNRS, Université Toulouse III-Paul Sabatier, Centre de Recherches en Cancérologie de Toulouse, INSERM U1037, 31037 Toulouse, France; 6 Barcelona Supercomputing Center, 08034 Barcelona, Spain; 7 Department of Hematology, University Hospital Essen, University of Duisburg-Essen, Essen, Germany; 8 Institute for Pathology, Christian-Albrechts-University Kiel and University Hospital Schleswig Holstein, Campus Kiel, Kiel, Germany; 9 Institut d’Investigacions Biomediques August Pi I Sunyer (IDIBAPS), University of Barcelona, 08036 Barcelona, Spain; 10 Institució Catalana de Recerca i Estudis Avançats (ICREA), 08010 Barcelona, Spain; 11 Department of Internal Medicine, University Hospital Essen, St. Josef-Krankenhaus, University Medicine Essen, Essen, Germany; 12 Present Address: Institute of Human Genetics, University of Ulm and University Medical Center Ulm, Ulm, Germany

**Keywords:** T-LGLL, Large granular lymphocytic leukemia, BCL11B, DNA methylation, Pyrosequencing

## Abstract

**Background:**

The molecular pathogenesis of T-cell large granular lymphocytic leukemia (T-LGLL), a mature T-cell leukemia arising commonly from T-cell receptor *αβ*-positive CD8^+^ memory cytotoxic T cells, is only partly understood. The role of deregulated methylation in T-LGLL is not well known. We analyzed the epigenetic profile of T-LGLL cells of 11 patients compared to their normal counterparts by array-based DNA methylation profiling. For identification of molecular events driving the pathogenesis of T-LGLL, we compared the differentially methylated loci between the T-LGLL cases and normal T cells with chromatin segmentation data of benign T cells from the BLUEPRINT project. Moreover, we analyzed gene expression data of T-LGLL and benign T cells and validated the results by pyrosequencing in an extended cohort of 17 patients, including five patients with sequential samples.

**Results:**

We identified dysregulation of DNA methylation associated with altered gene expression in T-LGLL. Since T-LGLL is a rare disease, the samples size is low. But as confirmed for each sample, hypermethylation of T-LGLL cells at various CpG sites located at enhancer regions is a hallmark of this disease. The interaction of *BLC11B* and *C14orf64* as suggested by in silico data analysis could provide a novel pathogenetic mechanism that needs further experimental investigation.

**Conclusions:**

DNA methylation is altered in T-LGLL cells compared to benign T cells. In particular, *BCL11B* is highly significant differentially methylated in T-LGLL cells. Although our results have to be validated in a larger patient cohort, *BCL11B* could be considered as a potential biomarker for this leukemia. In addition, altered gene expression and hypermethylation of enhancer regions could serve as potential mechanisms for treatment of this disease. Gene interactions of dysregulated genes, like *BLC11B* and *C14orf64*, may play an important role in pathogenic mechanisms and should be further analyzed.

**Supplementary Information:**

The online version contains supplementary material available at 10.1186/s13148-022-01362-z.

## Background

T-LGLL is a mature T-cell leukemia arising commonly from T-cell receptor (TCR) *αβ*-positive CD3^+^ memory cytotoxic T cells (CD8-positive T cells) [1]. Co-expression of CD57 is frequently observed [2]. Patients can present with severe neutropenia and/or anemia with transfusion dependency. In neutropenic patients, life-threatening recurrent infections are observed. Morbidity and mortality are mostly due to the accompanying cytopenias [3]. Lymphocyte infiltration of the liver, spleen, or the bone marrow is observed. Cytopenias are not mediated by bone marrow infiltration of leukemic cells but are related to immunological dysfunction [4]. T-LGLL often arises in the context of autoimmune diseases, such as rheumatoid arthritis occurring in approximately one-third of patients with T-LGLL [5]. The median age at diagnosis is 60 years and around 25% of adult patients are under 50 years old [6].

Many patients can be asymptomatic or exhibit an indolent clinical course. If treatment is necessary, immunosuppressive therapy is used as first-line treatment but does not represent a curative option. Allogeneic stem cell transplantation is currently the only curative option for patients not responding to conventional therapy [7]. The median overall survival was reported to be around nine to ten years [8].

In T-LGLL patients, insufficient response to activation-induced cell death leading to efficient apoptosis is considered a major pathogenetic mechanism driving leukemogenesis. Various pro-apoptotic and pro-survival signaling pathways, including FAS/FASL, JAK2/STAT3, NF-κB, RAS/MEK/ERK, and PI3K/AKT pathways, are frequently deregulated in T-LGLL [9].

However, the molecular pathogenesis of T-LGLL is still only partly understood. Recurrent numeric and/or structural chromosomal imbalances are rare in T-LGLL [10]. Only a few recurrent somatic mutations have been discovered. Activating somatic mutations in the SRC homology 2 (SH2) domain of the signal transducer and activator of transcription 3 (STAT3) gene have been identified in 23–40% of T-LGLL patients [11, 12]. In contrast to other mature T-cell lymphomas, mutations in the STAT5B gene have been reported only in 2% of T-LGLL cases [13]. No other recurrent mutations were detected in an exome sequencing study of six T-LGLL cases [14]. Recently, we identified recurrent non-synonymous alterations in the *TNFAIP3* tumor suppressor gene in 8% of T-LGLL cases, likely contributing to deregulated NF-κB activity in this entity [11]. Nevertheless, the genome of T-LGLL appears to be stable and the pathogenesis of this T-cell leukemia remains largely unknown.

Therefore, we aimed to characterize the epigenetic profile of T-LGLL by array-based DNA methylation profiling and compared it to that of non-neoplastic T cells of various maturation stages, e.g., naive, central memory, and effector memory T cells, to identify molecular events driving the pathogenesis of T-LGLL. To characterize which genomic regulatory elements show T-LGLL-specific changes in DNA methylation, we compared the differentially methylated loci between the T-LGLL cases and normal T cells with chromatin segmentation data of benign T cells from the BLUEPRINT project [15]. Moreover, we analyzed gene expression data of T-LGLL and benign T cells and validated the results by pyrosequencing in an extended cohort, including five patients with sequential samples.

## Results

### DNA methylation signatures segregate T-LGLL cells from their benign counterpart

**Fig. 1:**
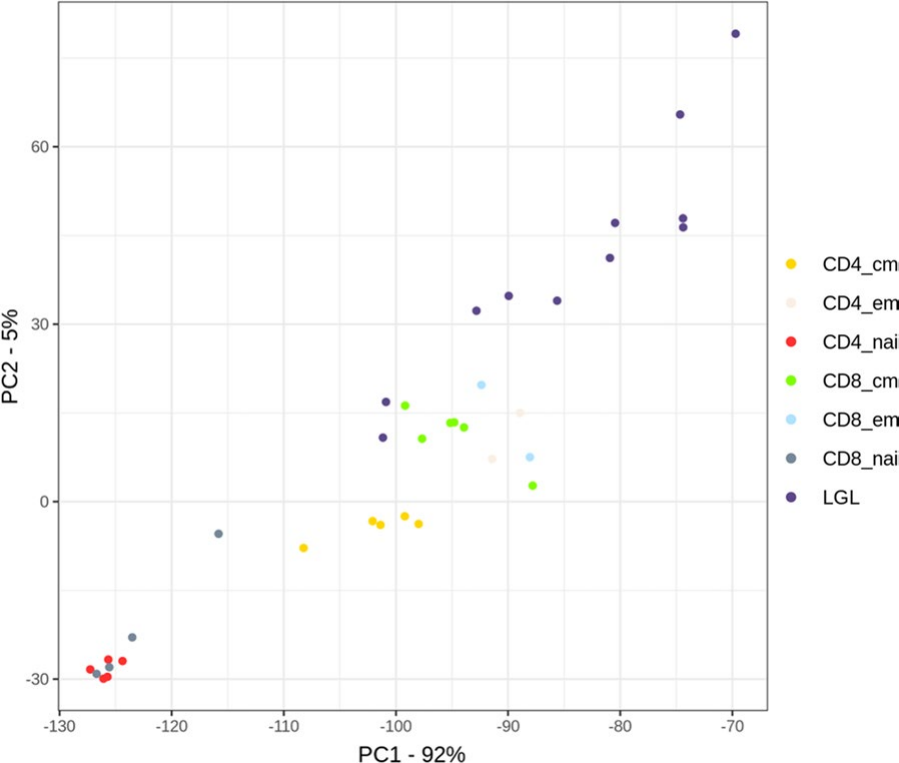
Genome-wide methylation signature of T-LGLL compared to normal T-cell subsets. Unsupervised Principal Component Analysis (PCA) of T-LGLL samples (LGL = purple) and benign T-cell subsets (CD4^+^ central memory = yellow; CD4^+^ effector memory = rose; CD4^+^ naïve = red; CD8^+^ central memory = green; CD8^+^ effector memory = blue; CD8^+^ naïve = gray) of the top two components. Beta-values of 39,930 CpG loci with the highest variation (sd/sd_max>_ = 0.4) are visualized

Since the DNA methylation signature varies according to tissues and cell types [16, 17], identifying appropriate non-neoplastic samples for comparison is crucial for detecting de novo DNA methylation changes in tumor samples. We performed genome-wide DNA methylation profiling of FACS-sorted tumor cell subsets from eleven T-LGLL cases. Six subsets of T cells of healthy donors, including CD4^+^ naïve cells, CD4^+^ central memory and CD4^+^ effector memory cells, CD8^+^ naïve cells, CD8^+^ central memory, and CD8^+^ effector memory cells were used for the comparison to non-neoplastic human T-cell subsets. We compared the malignant T-LGLL samples to the mature T-cell subsets, since it is thought that the malignant T-LGLL cells arise from mature T cells. Unsupervised Principal Component Analysis (PCA) of all normal T-cell subsets and leukemia samples based on the 39,930 CpG loci with the highest variation in beta-values (sd/sd_max>_ = 0.4) clearly segregated the dataset into the different subsets of normal and malignant T cells (Fig. 1). Both principal components (PC1 (92% of variance) and PC2 (5% of variance)) separate T-LGLL samples from benign T cells and the T-LGLL cells cluster closest to CD8-positive effector and central memory T cells. Due to this pattern and previous literature about the cell of origin of T-LGLL cells, we decided to primarily compare the T-LGLL samples to CD8-positive memory T cells, which is the one also closest to the described cell of origin of T-LGLL [18]. Thus, we compared the DNA methylation profile of 11 T-LGLL (for patient characteristics, see Additional file 1: Table S1) to eight non-neoplastic CD8-positive memory T-cell samples of healthy donors.

**Fig. 2:**
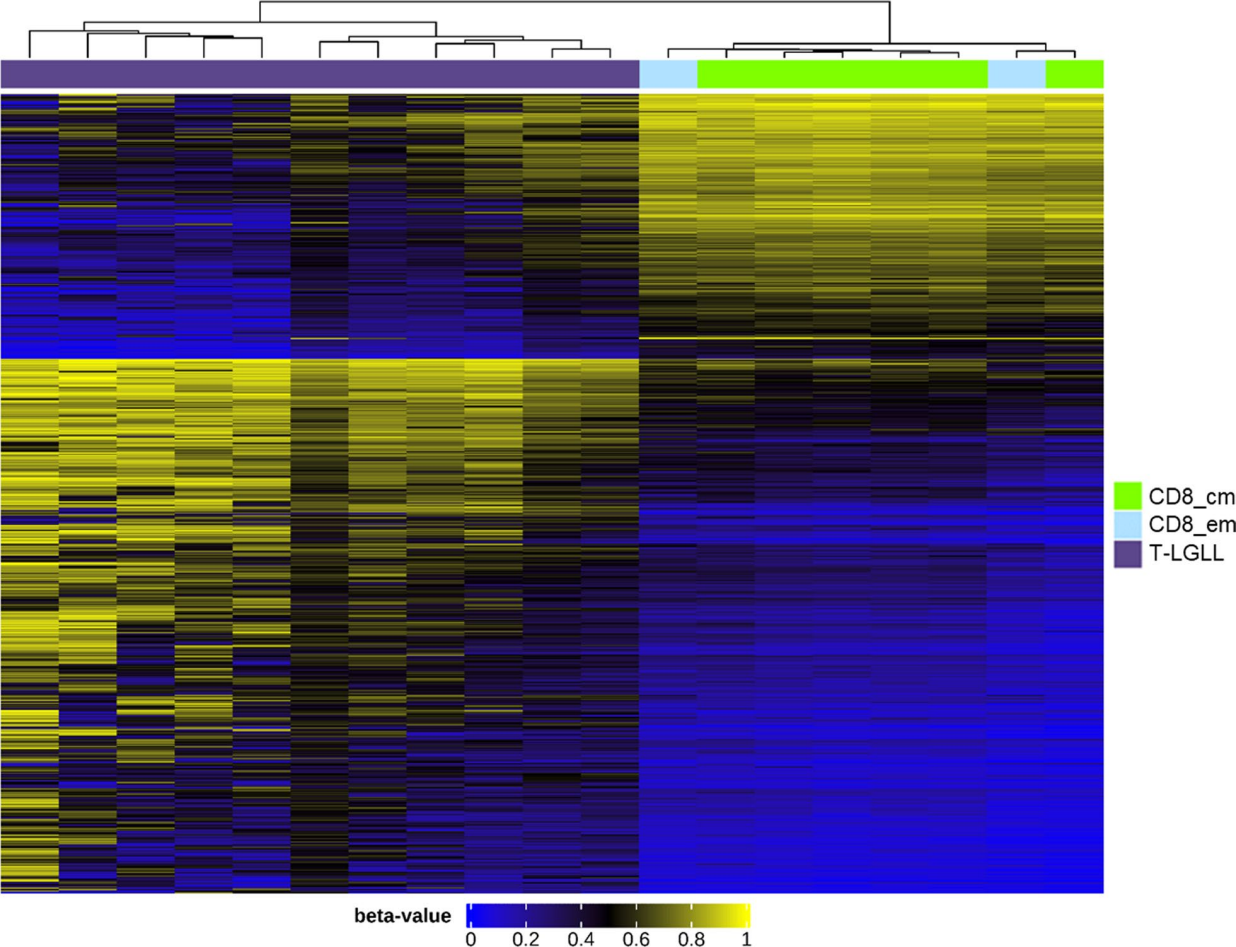
Genome-wide methylation signature of T-LGLL and CD8^+^ memory cells. Heatmap of supervised cluster analysis of T-LGLL samples (purple) and CD8 positive memory cells (CD8^+^ central memory = green; CD8^+^ effector memory = blue), using adjusted *p* value < 0.005 and delta Beta > 0.25 lead to visualization in this heatmap of 2216 highly differentially methylated CpG loci

Two thousand two hundred and sixteen CpG probes showed an absolute differential methylation between T-LGLL and CD8-positive memory samples of over 25% (delta > 0.25). Of these, 1479 CpG loci were hypermethylated and 737 were hypomethylated in T-LGLL samples in comparison with the normal T cells (Fig. 2). None of them were probes interrogating Ch-loci (non-CpG loci). Differentially methylated positions (DMPs) were annotated with matching genomic features (Additional file 1: Table S9).

### Identification of differentially methylated gene regions and candidate genes

We annotated the 2216 DMPs to identify candidate genes potentially involved in the pathobiology of T-LGLL. Then, we ranked the genes according to the proportion of significant DMPs for each gene, taking into account the methylation status (hyper or hypo) of CpGs. We found 267 genes hypermethylated and 90 genes hypomethylated in T-LGLL samples (Additional file 1: Tables S10 and S11). Among the hypermethylated genes were *BCL11B*, *TTC39C*, *THEMIS,* and *NKX6-2* (Additional file 1: Table S10) and the hypomethylated genes included *ZEB2*, *MAST3*, *RIN3*, *SLC15A4,* and *FAM53B* (Additional file 1: Table S11).

We additionally performed a methylation differential analysis by region to find candidate genes. This has become of interest lately as it has been described to be associated with phenotypic changes in cancer [19, 20]. We found a total of 1096 differentially methylated regions (DMRs) with lengths from 3 to 5321 bp containing between 2 and 70 CpGs (Additional file 1: Table S12). Ordering DMRs by the mean difference of beta-values between the T-LGLL and normal T-cell samples, we found the most hypermethylated regions in the genes *BCL11B*, *TNRC6B*, *TTC39C*, *THEMIS,* and *LYN*; and the most hypomethylated regions in the genes *MEF2A*, *RIN3*, *ZEB2*, *DENND3*, *RFC2,* and *RUNX3*.

Teramo et al*. *[21] have reported SOCS3 being down-modulated in T-LGLL. We therefore tested methylation differences in CpG loci of the *SOCS3* promoter. We did not observe significant differences between T-LGLL and healthy donor-derived CD8 memory cells, but in some cases a trend was visible for promoter CpG hypermethylation related with downregulated expression (Additional files 2 and 3: Fig. S2).

### Functional enrichment analysis reveals enrichment in pathways related to immune response

We hypothesized that genes with significant changes in DNA methylation in promoters and enhancers will constitute most of the functional biological change associated with the CpG hyper- or hypomethylation. In order to find the most relevant functional changes, we performed a gene ontology analysis on genes hyper- and hypomethylated in promoters and enhancers. These genes were selected from hyper- and hypomethylated genes shown in Additional file 1: Tables S10 and S11, selecting those with *UCSC_RefGene_Group* assigned to *TSS200* (200 bp upstream the transcription start site, TSS) or *5’UTR* or assigned to *Enhancer* category in the 450 k annotation (Additional file 1: Tables S13–S16).

A statistical enrichment (*p* value < 0.01) was observed in promoters of hypermethylated genes in pathways related to the immune response, especially from T cells (Additional files 1 and 4: Table S15 and Fig. S3A). For hypomethylated genes, promoter pathways were also enriched in immune pathways, although more related to humoral responses (Additional files 1 and 5: Table S16 and Fig. S3B).

### Mapping of differentially methylated CpGs and regions reveals enrichment of hypomethylated and hypermethylated loci in active enhancer sites

**Fig. 3:**
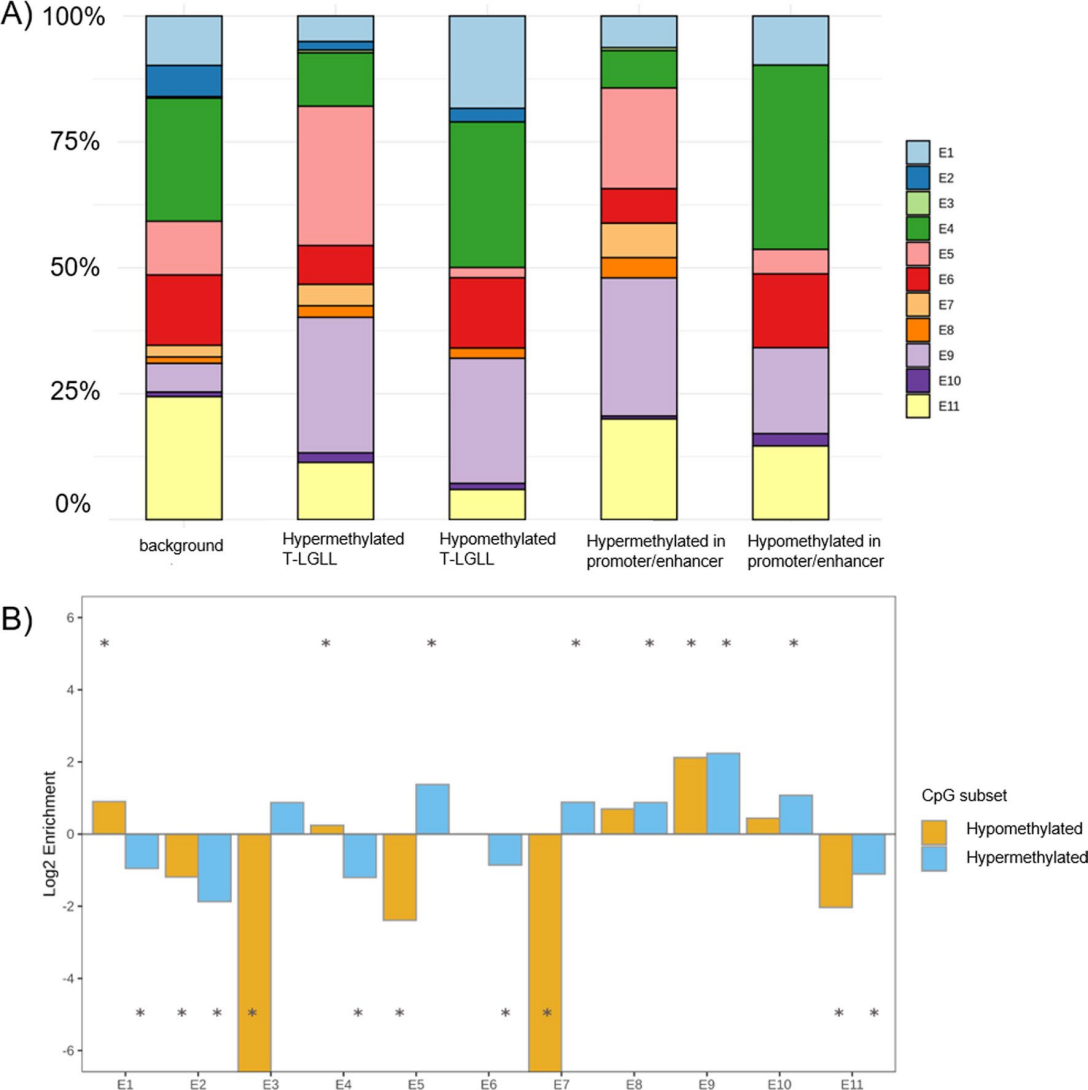
Annotation of differentially methylated CpG loci in T-LGLL with chromatin states of CD8^+^ memory cells. **A** Differentially hyper- or hypomethylated CpG loci between T-LGLL and normal T-cell subsets were annotated by the chromatin segmentation data of CD8^+^ memory cells from the BLUEPRINT project (chromatin segmentations E1–E11 are depicted in different colors in %, color code explained in Additional file 1: Table S17). The first “background” bar represents the loci of the array. From left to right the following CpG subsets are shown: (1) All 450 k CpGs, annotated to chromatin states (2) Significant hypermethylated CpGs in T-LGLL samples vs non-neoplastic T cells, (3) significant. hypomethylated CpGs in T-LGLL samples vs non-neoplastic T cells, (4) significant hypermethylated CpGs located in promoters or enhancers (as defined by the Illumina 450 k annotation, see Additional file 11) in T-LGLL samples vs non-neoplastic T cells, (5) significant hypomethylated CpGs located in promoters or enhancers (as defined by the Illumina 450 k annotation, see methods) in T-LGLL samples vs non-neoplastic T cells. **B** Chromatin state enrichment of CD8-positive memory genome regions containing significantly differentially methylated CpG loci (hyper and hypomethylated, bar 2 and 3, Fig. 3A, respectively) compared to 450 k background CpG loci (bar 1, Fig. 3A). (*) Significant enrichments (hypergeometric test, *p* value < 0.01). Chromatin segmentation E1–E11 is described in Additional file 1: Table S17

We mapped the differentially methylated CpGs from T-LGLL samples to the chromatin states determined in CD8-positive memory cells by the BLUEPRINT/IHEC Project (Additional file 1: Tables S7 and S17). T-LGLL hypomethylated and hypermethylated loci were enriched in active enhancer sites (“E9” state, 4.35- and 4.71-fold change, *p* = 2.52 × 10^–65^ and *p* = 1.99 × 10^–152^, respectively, Fig. 3A and 3B, Additional file 1: Table S13) defined by the activating marks H3K4me1 and H3K27Ac. A similar enrichment was found when considering significant CpGs in genes with hyper- or hypomethylated promoters/enhancers, as defined in the previous section (“E9” state, 2.99- and 4.80-fold change, *p* = 2 × 10^–3^ and *p* = 6.46 × 10^–21^, respectively, Additional file 1: Tables S15 and S16).

### Gene expression analysis by qPCR reveals significant differences in candidate genes between T-LGLL and healthy donor-derived CD8^+^ T cells

We analyzed transcript levels of nine genes by qPCR to identify whether significantly differential methylation at CpG loci correlates with gene expression differences of candidate genes. *GAPDH* was used as a housekeeping gene (Additional file 1: Table S8). The selection of genes was based on (a) the most frequent differentially methylated genes and (b) genes of interest known to be relevant in pathways involved in T-cell development or lymphomagenesis.

**Fig. 4:**
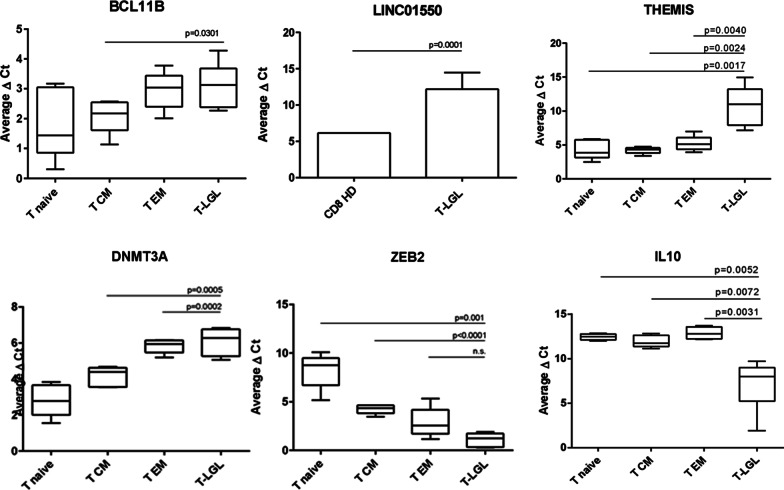
Expression analysis of T-LGLL samples compared to normal T-cell subsets. Expression analysis (qPCR) of T-LGLL samples of indicated genes identified as hyper- or hypomethylated compared to healthy donor-derived samples (CD8^+^ T-cell subsets: T naïve = CD8^+^ naïve cells; T CM = CD8^+^ central memory cells; T EM = CD8^+^ effector memory cells; T-LGLL = T-LGLL samples; CD8 HD = healthy donor bulk CD8^+^ cells). High average delta CT values indicate low expression

In the group of hypermethylated genes, *BCL11B*, *THEMIS, C14orf64* (alternative name *LINC01550*), and *DNMT3A* were analyzed using qPCR. All four genes exhibited a significant lower expression in T-LGLL compared to normal CD8 memory cells. For *BCL11B* expression differences were significant only for CD8 central memory cells. For the long intergenic non-protein coding (LINC) *LINC01550,* bulk CD8^+^ T cells were available for comparison. Gene expression of this gene in T-LGLL was significantly lower compared to the benign T cells (Fig. 4).

In the group of hypomethylated genes, *ZEB2, IL10, MAST3,* and *PTEN* were analyzed. *ZEB2* and *IL10* displayed a higher expression in T-LGLL cells than in normal CD8^+^ T cells (Fig. 4)*. MAST3* and *PTEN* did not show any significant differences in gene expression levels. We also studied *IL6*, as the corresponding protein is known to be highly expressed in T-LGLL. We could confirm significantly higher *IL6* levels in T-LGLL compared to CD8-positive T cells from healthy donors (Additional file 6: Fig. S4).

In summary, we observed significant differences in the gene expression of hyper- and hypomethylated genes between T-LGLL cells and healthy donor-derived memory T cells.

### BCL11B is strongly epigenetically changed in T-LGLL cells

Remarkably, among the top eight hypermethylated regions four were located in *BCL11B* (mean difference in beta-values of 0.78, 0.68, 0.52, 0.47) (Additional file 1: Table S10). 21 CpGs—from a total of 62 interrogated—annotated to this gene were found as DMPs with an FDR of ≤ 0.5%, all of them hypermethylated in T-LGLL samples (Additional file 1: Table S9). The four significant DMRs were located in the gene body and assigned as enhancers by ENCODE (Additional file 1: Tables S10 and S15), and these match enhancers in CD8-positive memory cells (Additional files 7 and 8: Fig. S5A and S5B). Furthermore, expression analysis using qPCR (Fig. 4) showed a significant decrease in *BCL11B* expression in T-LGLL samples compared to CD8-positive central memory T cells.

### Hypermethylated CpGs in BCL11B and C14orf64 are epigenetic hallmarks of T-LGLL

**Fig. 5:**
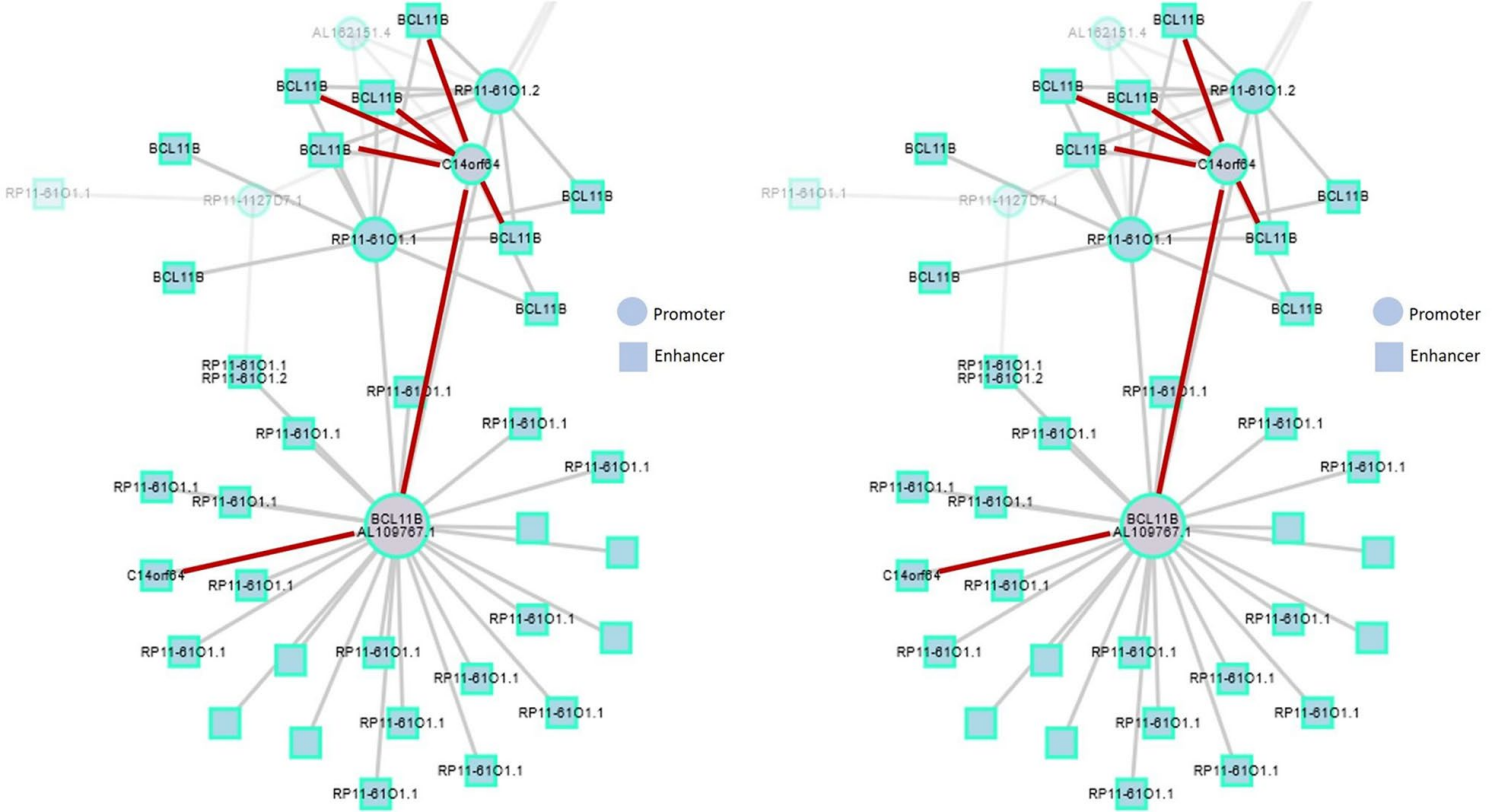
Interaction of the gene *BCL11B* with other genes in CD8^+^ cells. BCL11B long-range interactions from Promoter Capture Hi-C for total CD8-positive T cells (red: BCL11B–C14orf64 (LINC01550) interactions). Genomic regions are depicted in blue circles (promoter region) or blue square (enhancer region)

Exploratory analysis in promoter capture Hi-C data to identify long-range interacting regions of 31,253 promoters in 17 human primary hematopoietic cell types from the BLUEPRINT/IHEC Project [15] showed a promoter—promoter interaction between *BCL11B* and *C14orf64* in CD8-positive T cells (see Fig. 5). Interestingly, the long intergenic non-coding RNA *C14orf64* shows a highly hypermethylated region in our analysis (mean difference in beta-values of 0.32) (Additional file 1: Table S9). *BCL11B* and *C14orf64* both showed lower expression in T-LGLL. The close interaction in the nucleus of these genes in CD8-positive T cells and the apparent coordination of their regulation by methylation mechanisms in T-LGLL suggest a potentially relevant role for the pathogenesis and clinical behavior of T-LGLL cells.

**Fig. 6:**
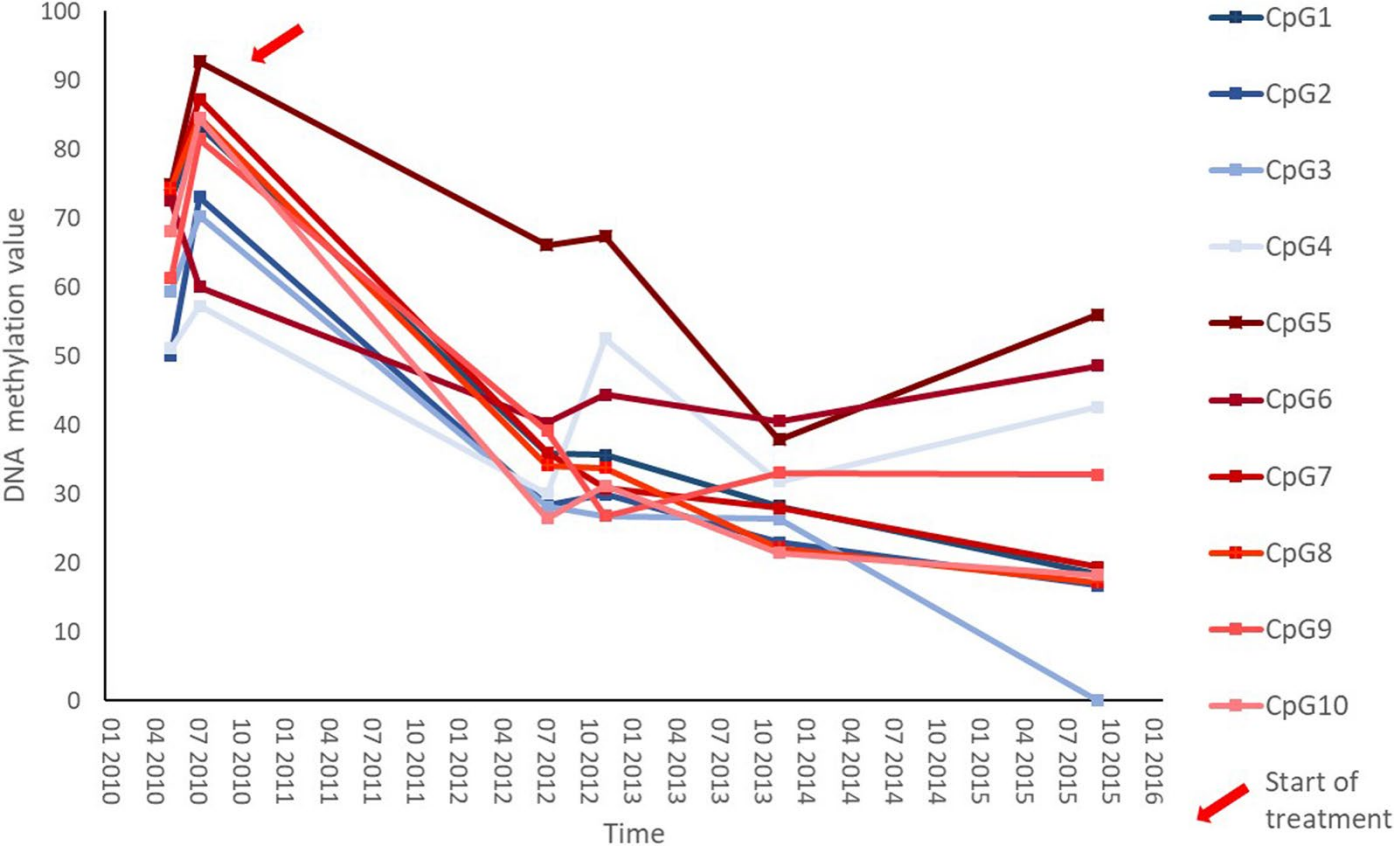
Methylation values over time for ten CpG sites in “patient 2” determined by bisulfite pyrosequencing. Response to therapy is indicated by descending methylation values. Sampling was not performed in a linear manner

To validate the results, we performed pyrosequencing on selected CpG loci of *BCL11B* and *C14orf64* to estimate the potential of these genes as DNA methylation biomarkers (Additional file 1: Table S18). We selected six highly differentially methylated CpG sites located at enhancer sites of *BCL11B* and four CpG sites of *C14orf64*. DNA methylation levels of these ten CpGs were measured by BPS in all T-LGLL samples and three normal T-cell samples with sufficient material. High correlation was observed between pyrosequencing and methylation arrays as measured by bivariate correlation analysis (average Pearson’s coefficient of 0.95, *p* < 0.001) for each CpG site (Additional files 1 and 9: Table S6 and Fig. S1). Changes of DNA methylation level at these ten CpG sites (Additional file 1: Table S5) were evaluated during the course of disease with and without treatment in five T-LGLL patients. We applied the BPS analysis of the ten CpG sites to samples obtained at three to six different time points. One out of five patients (patient 2) achieved a complete remission while the other patients presented active disease during the follow-up period and had high methylation values in the ten CpGs assayed (Additional file 1: Table S19). Interestingly, the only patient exhibiting a complete response showed a decrease in the methylation values after initiation of therapy, in line with response to therapy (Fig. 6).

## Discussion

The cell of origin in CD8-expressing T-LGLL is assumed to be a terminal effector-type cytotoxic T cell [18]. As analyzed by transcriptome sequencing and gene expression arrays, these cells are characterized by down-modulation of CD28 and overexpression of perforin, granzymes, and CD57, which is in line with their cytotoxic phenotype [18]. Our PCA of array-based DNA methylation profiling of CD8-positive T-LGLL samples and healthy donor-derived T cell subsets revealed the closest similarity between T-LGLL and CD8^+^ memory T cells. Based on these methylation analyses, our results confirm this subset as the non-neoplastic counterpart as described before by transcriptomic analyses [18]. DNA methylation analyses did not allow further differentiation between CD8 central memory and/or effector memory T cells, because PCA revealed a homogeneous clustering of these two subsets and differential methylation analyses of these subsets did not reveal any significant differences.

Only few publications report on methylation changes in human T-LGLL [21] and no comprehensive methylation profiling has been reported until now. Mapping of differentially methylated CpGs between T-LGLL and the non-neoplastic counterpart revealed major differences in methylation profiles, pointing to an important role for aberrant methylation in this disease.

In the group of differentially hypermethylated genes, the genes *BCL11B* and *THEMIS* were among the genes with the highest ratio of differentially methylated CpGs. The BAF chromatin remodeling complex subunit *BCL11B* gene is located on chromosome 14q32.2. The encoded protein, B-cell lymphoma/leukemia 11B, functions as a C2H2 zinc finger transcription factor in early T cell development [22]. Its specific role in human mature T cells is not fully understood and needs further investigation. It is known to act as a tumor suppressor gene in various malignancies, while in some cases it has been reported to have oncogenic features [23, 24]. Low expression of BCL11B has been associated with poor prognosis in T-acute lymphoblastic leukemia [25]. Hypermethylated *BCL11B* showed significantly lower mRNA expression in T-LGLL compared to normal CD8^+^ T cells, likely contributing to disease promotion.

*THEMIS*, Thymocyte Selection Associated, located on chromosome 6q22.33, encodes a protein that regulates T cell selection during late thymocyte development and is necessary for lineage commitment and maturation of T cells. More specifically, in mice Themis controls lineage selection of double positive thymocytes into either CD4- or CD8-positive T cells [26]. Post-selection deletion of Themis reduces the homeostatic response of peripheral CD8-positive T cells to self-peptide–major histocompatibility complex in mice [27]. Its role in T-cell leukemia/ lymphoma is largely unknown, but THEMIS was recently shown to be downregulated by promoter methylation in HTLV-1-infected CD4-positive cells of adult T-cell leukemia [28]. Importantly, we also observed hypermethylation of *THEMIS* in T-LGLL resulting in its downregulation at the transcriptomic level. In aggregate, these findings suggest that epigenetic deregulation of *THEMIS* may play a common role in the molecular pathogenesis of different T-cell neoplasms and thus deserves further investigation. Furthermore, our data provide a rationale for testing hypomethylating agents in T-LGLL.

Analyzing the most significantly hypomethylated genes, *IL10* and *ZEB2* were of particular interest. IL10, produced by monocytes and lymphocytes, is involved in the regulation of the JAK/STAT pathway. Binding of IL10 to its receptor leads to phosphorylation of STAT3 and hence activates the JAK/STAT pathway [29]. IL10 signaling is known to induce SOCS3, a negative regulator of the JAK/STAT pathway [30, 31]. SOCS3 is known to be epigenetically silenced in JAK/STAT-dependent tumors [32, 33] and can be induced by IL6, for which very high levels in T-LGLL are described [21]. Teramo et al*.* have reported SOCS3 being unresponsive to IL6 triggering in T-LGLL. IL6 mediated SOCS3 expression could be restored by application of demethylation agents leading to apoptosis of T-LGLL cells [21]. Testing of methylation differences in CpGs of the SOCS3 promoter revealed no significant differences between T-LGLL and healthy donor-derived CD8 memory cells, although variability between subjects can be observed (Additional files 2 and 3: Fig. S2). This can be explained by using different methods and statistics in the methylation analyses that tend to homogenize differences between groups hiding variability between subjects. The role of SOCS3 methylation as a hallmark for treatment personalization should be analyzed in further studies. In line with hypomethylation of *IL10* in T-LGLL, we identified a significant overexpression of IL10 at the mRNA level compared to healthy donor-derived CD8 memory cells, pointing to a further potentially novel mechanism of JAK/STAT-activation in T-LGLL.

ZEB2, Zinc Finger E-Box Binding Homeobox 2, among others, regulates the terminal differentiation of CD8-positive cytotoxic T cells [34]. It is overexpressed in several tumor entities including T-cell malignancies where it functions as an oncogene [35, 36]. We confirmed overexpression of *ZEB2* by qRT-PCR in T-LGLL samples pointing to a potential oncogenic role of this gene also in T-LGLL.

Serial analyses of *BCL11B* methylation in individual patients suggest that methylation changes in T-LGLL appear to be stable over time. Disappearance of clonal T-LGLL cells in response to successful treatment with immunosuppressive agents resulted in the normalization of methylation states (Fig. 6). Together, this observation suggests that hypermethylation of *BCL11B* may be considered a novel molecular hallmark of T-LGLL and corresponds to the tumor cell content. To confirm, that monitoring of *BCL11B* methylation could be used both as a marker in the diagnostic process and for monitoring minimal residual disease, the validation in a larger cohort with sequential sampling of T-LGLL patients is needed.

Furthermore, our data in combination with in vitro studies reported by Teramo and colleagues [21] provide a rationale for the development of novel epigenetic treatment modalities in T-LGLL employing demethylating agents.

Array-based analysis of the overall methylation profile of T-LGLL samples in comparison to healthy donor-derived CD8-positive T cells showed that differentially methylated loci were enriched in enhancer sites whereas hypomethylation was not present in repressed polycomb promoter regions. These results suggest that regulation of gene expression by DNA methylation changes is acting through the modulation of enhancers. Compared to benign CD8-positive T cells, heterochromatin-typical H3K27me3 states are enriched in T-LGLL with hypermethylated regions and depleted in T-LGLL with hypomethylated regions including promoters. H3K27me3-rich genomic regions can function as silencers to repress gene expression via chromatin interactions, e.g., proximity or looping [37]. H3K27me3 is known to be accumulated in promoters of many cancer types, where it is associated with gene repression [38] and a poor clinical outcome [39].

Methylation patterns of T-LGLL patients showed a regulation of BCL11B expression most likely through methylation of the gene. qPCR revealed low expression of this gene in T-LGLL compared to its non-neoplastic counterpart. Low BCL11B expression was an independent indicator for shorter overall survival and time to recurrence for patients with hepatocellular carcinoma [40]. BCL11B has been confirmed as a tumor suppressor in hepatocellular carcinoma with inhibitory effects on proliferation, cell cycle progression, apoptosis, and mobility [40]. In our analysis, aberrantly methylated CpGs were not located in the promoter but in the gene body in introns 2 and 3, a region in which an enhancer was identified based on chromatin annotation data from CD8 memory cells. Previously published data on murine Bcl11b studies have revealed DMRs in the 5′-flanking region, identified as both T cell-specific enhancer and silencer. Introns 1 and 3 were supposed to contain cis-regulatory elements [41], underlining the role of intronic regulatory elements in the regulation of this gene.

Identification of long-range interacting regions by promoter capture Hi-C data from the BLUEPRINT/IHEC Project [15] on CD8-positive T cells showed a promoter—promoter interaction between the genomic regions of *BCL11B* and *C14orf64* (*LINC01550*) (Fig. 5). *C14orf64* is a long intergenic non-protein coding RNA, the overexpression of which is known to be associated with poor survival in lung adenocarcinoma patients [42]. By contrast, elevation of LINC01550 induces apoptosis and cell cycle arrest leading to a better outcome of patients with malignant melanoma [43]. We identified a highly hypermethylated region in this LINC RNA (Additional file 1: Table S6). Visualization of the 3D chromatin contacts displayed as a network revealed an interaction of the promoters of *BCL11B* and *LINC01550* in the nucleus of CD8-positive cells. We observed a high correlation between both genes based on expression in many cell types (Additional file 10: Fig. S6). The apparent coordination of their expression regulated by methylation mechanisms in T-LGLL suggests a relevant role for the pathogenesis and clinical behavior of the disease.

## Conclusions

We identified dysregulation of DNA methylation associated with altered gene expression in T-LGLL. Since T-LGLL is a rare disease, the samples size is low and monitoring of *BCL11B* methylation in more sequential T-LGLL patient samples is needed. Hypermethylation of T-LGLL cells at various CpG sites located at enhancer regions is a hallmark of this disease. The interaction of *BLC11B* and *C14orf64* as suggested by in silico data analysis could provide a novel pathogenetic mechanism that needs further experimental investigation.

## Material and methods

### Patient cohort and non-neoplastic human T-cell samples

Seventeen T-LGLL patients with high-quality DNA extracted from sorted tumor cells were included in this study. The mean tumor cell content after tumor cell enrichment was 96% (range 95–98%) as determined by FACS. For eleven T-LGLL cases, genome-wide DNA methylation profiling was performed at diagnosis. Results were confirmed by pyrosequencing in all 17 patients; for five patients, sequential samples were available during their clinical course. Patient characteristics and sample overview are described in Additional file 1: Tables S1 and S3. Gene expression analysis was performed on leukemic cells of eleven patients, depending on material availability. Samples of 16 healthy donors were included for comparison, six of them in DNA methylation profiling, three in pyrosequencing, and ten in the gene expression analysis. Non-neoplastic TCR *αβ*^+^ T cell subsets were isolated from healthy donors, matching age and gender. Healthy blood donors of benign T cells sorted for methylation array analysis were anonymized. For two healthy donors, only bulk CD8 T cells were available for gene expression analysis. Cell isolation information for non-neoplastic samples and a sample overview are described in Additional file 1: Tables S2 and S3.

The diagnosis of T-LGLL was based on (a) flow cytometry analyses from peripheral blood (PB) showing > 400/µl of CD3^+^CD8^+^CD57^+^ cells, and (b) evidence of clonal TCR gene rearrangements.

### Sample preparation and sorting of T cell subsets

PB mononuclear cells were isolated by Pancoll density centrifugation (PAN-Biotech, Aidenbach, Germany). Leukemic cells were isolated as CD3^+^CD8^+^CD57^+^ T cells by fluorescence-activated cell sorting (FACS, ARIA III, BD Biosciences; Heidelberg, Germany). T cells from healthy donors were initially enriched (> 98% purity) for either CD4^+^ or CD8^+^ T cells by magnetic cell separation using the MACS system (Miltenyi Biotec, Bergisch Gladbach, Germany). Afterward, the T cell subsets from healthy donors were isolated by FACS using the following strategies: CD8 naïve T cells: CD8^+^CD45RA^+^CD27^+^, CD8 central memory T cells: CD8^+^CD45RA^−^CD27^+^, CD8 effector memory T cells: CD8^+^CD45RA^−^CD27^−^, CD4 naïve T cells: CD4^+^CD45RA^+^CD27^+^, CD4 central memory T cells: CD4^+^CD45RA^−^CD27^+^, and CD4 effector memory T cells: CD4^+^CD45RA^−^CD27^−^. FACS confirmed a 90–99% purity of the T cell subsets. The following antibodies were used: anti-CD3-PerCP, anti-CD4-PE, anti-CD8-APC-Cy7, anti-CD27-APC, and anti-CD45RA-FITC (all Miltenyi Biotech). Genomic DNA was extracted from all samples using the DNeasy Blood & Tissue Kit (Qiagen, Hilden, Germany).

Analysis of differential gene expression by reverse transcription real-time PCR (qPCR) and Pyrosequencing is described in Additional file 11.

### Array-based DNA methylation analysis

Bisulfite conversion was performed using the EZ-DNA Methylation Kit^®^ (Zymo Research Corporation, Irvine, CA, USA), followed by DNA methylation analysis with the Infinium HumanMethylation450 BeadChip (Illumina, Inc., San Diego, CA, USA). All arrays were successfully scanned using the Illumina iScan^TM^System (more detailed description in: [44]. To identify suitable non-neoplastic samples for eleven T-LGLL samples, we included 24 T cell samples sorted from PB of six healthy blood donors. These included: CD4^+^ naïve T cells (CD4nai, *n* = 5), CD4^+^ central memory T cells (CD4 + cm, *n* = 5), CD4^+^ effector memory T cells (CD4 + em, *n* = 2), CD8^+^ naïve T cells (CD8-positive nai, *n* = 4), CD8^+^ central memory T cells (CD8-positive cm, *n* = 6), and CD8^+^ effector memory T cells (CD8-positive em, *n* = 2). After normalization and filtering of poor-quality probes, multi-mappers, probes in sex chromosomes, and single nucleotide polymorphisms, beta-values of 401,778 CpG sites were subjected to differential analysis between CD8-positive memory and T-LGLL samples, resulting in 11,375 differentially methylated loci (adjusted *p* value < 0.005). For statistical analyses of the DNA methylation data and Functional enrichment analysis, see Additional file 11.

### Annotation of chromatin segmentation

All CpG sites that passed the filter for analysis (401,778) were annotated according to the chromatin state categories defined for effector memory CD8-positive, alpha–beta T cell data from the BLUEPRINT project (sample code C003UQ; Venous blood, male) (http://ftp.ebi.ac.uk/pub/databases/blueprint/). For that purpose, genome segmentation by chromatin states was performed in this sample. Briefly, chromatin immunoprecipitation (ChIP)-seq data for H3K4me3, H3K4me1, H3K36me3, H3K27Ac, H3K27me3, and H3K9me3 from samples were used to segment the genome of the respective cell types into defined chromatin states. Segmentation was performed with the ChromHMM package (v1.10; [45]) using an 11 state combinatorial chromatin model (Additional file 1: Table S7) (defined by the BLUEPRINT consortium for 20,150,128 release:

ftp://ftp.ebi.ac.uk/pub/databases/blueprint/paper_data_sets/chromatin_states_carrillo_build37). Each CpG position was located within a region with a specific chromatin state assigned. Significant differentially methylated CpGs were compared to all background CpGs in the different chromatin states. To calculate enrichment of the significant CpG dataset (hyper- or hypo-) for each chromatin state, we performed a hypergeometric test using the *base* package in *R*, considering significant enrichments with *p* value < 0.01.

### Promoter capture Hi-C sequencing data

CD8-positive T cell data from promoter capture Hi-C experiments were obtained from Javierre et al. [46], details on data processing and analysis can be accessed in the material and methods section. Only those interactions with a threshold above five from their analysis were considered, following the authors’ recommendation. GARDEN-NET was used to visualize BCL11B long-range interactions for total CD8-positive T cells [47].

## Supplementary Information

**Additional file 1: Table S1.** T-LGL patient data. **Table S2.** Benign samples. **Table S3.** Sample overview. **Table S4.** Primer sequences and CpGs used in pyrosequencing. **Table S5.** CpGs validated by pyrosequencing. **Table S6.** Pearson correlation of results of bisulfite sequencing and methylation array data. **Table S7.** Chromatin states—description. **Table S8.** Taqman probes used in qPCR. **Table S9.** Differentially methylated probes and genomic features. **Table S10.** Differentially hypermethylated genes. **Table S11.** Differentially hypomethylated genes. **Table S12.** Differentially methylated regions. **Table S13.** Gene ontology analysis for TSS200 or 5’UTR of hypermethylated genes identified in Table S10. **Table S14.** Gene ontology analysis for TSS200 or 5’UTR of hypomethylated genes identified in Table S11. **Table S15.** Gene ontology analysis for enhancers of hypermethylated genes identified in Table S10. **Table S16.** Gene ontology analysis for enhancers of hypomethylated genes identified in Table S11. **Table S17.** Chromatin states. **Table S18.** Pyrosequencing—raw data. **Table S19.** Sequential Samples—raw data.

**Additional file 2: Fig. S2.** Differential methylation of CpG loci in the *SOCS3* promoter in T-LGL samples. Comparison of CpG methylation (beta-value) for CpGs in the SOCS3 promoter between CD8+ memory cells (CD8mem) and T-LGLL samples (LGL). On top, adjusted p val of differential methylation analysis (dmpFDR, top left) and adjusted *p* value of differential variability analysis (dmVar, top right).

**Additional file 3: Fig. S2.** Differential methylation of CpG loci in the SOCS3 promoter in T-LGL samples. Comparison of CpG methylation (beta-value) for CpGs in the SOCS3 promoter between CD8+ memory cells (CD8mem) and T-LGLL samples (LGL). On top, adjusted p val of differential methylation analysis (dmpFDR, top left) and adjusted *p* value of differential variability analysis (dmVar, top right).

**Additional file 4: Fig. S3A.** Gene Ontology analysis of genes hyper- (A) and hypomethylated (B) of T-LGL patients. Significant Biological processes (GO database) enriched in genes associated with significantly differentially methylated CpG loci in T-LGL. Enrichment represented as odds ratio. Point size represents the gene count of each pathway. Enrichment *p* value obtained by overrepresentation analysis [30], represented by point color. **A** Gene Ontology analysis of hypermethylated genes in T-LGL.

**Additional file 5: Fig. S3B.** Gene Ontology analysis of genes hyper- (A) and hypomethylated (B) of T-LGL patients. Significant Biological processes (GO database) enriched in genes associated with significantly differentially methylated CpG loci in T-LGL. Enrichment represented as odds ratio. Point size represents the gene count of each pathway. Enrichment *p* value obtained by overrepresentation analysis [30], represented by point color. **B** Gene Ontology analysis of hypomethylated genes in T-LGL.

**Additional file 6: Fig. S4.** Differential gene expression of *IL6* between T-LGL and healthy donor-derived CD8+ memory T cells. Differential gene expression for *IL6* was measured by qPCR. Bulk CD8+ cells from two healthy donors were used for comparison. In line with previous publications, the T-LGLL cohort analyzed exhibits a higher *IL6* expression compared to healthy donor-derived C8+ cells. HD Healthy donor.

**Additional file 7: Fig. S5A.** (A and B): Location of differentially methylated CpG loci in the genes *BCL11B* and *C14orf64* (*LINC01550*). **A** Significant differentially methylated CpGs in BCL11B (T-LGLL compared to CD8+. memory T cells) were located in the gene body and assigned as enhancers by ENCODE, which match as enhancers in CD8-positive memory cells.

**Additional file 8: Fig. S5B.** (A and B): Location of differentially methylated CpG loci in the genes *BCL11B* and *C14orf64* (*LINC01550*). **B** Significant differentially methylated CpGs in *C14orf64* (*LINC01550*) (T-LGL compared to CD8 pos. memory T cells).

**Additional file 9: Fig. S1.** Correlation of bisulfite Pyrosequencing (BPS) and methylation Array DNA methylation levels. The correlation matrix shows the Pearson correlation coefficient (*r*: 1 (red) to − 1 (blue) among all CpG loci analyzed by BPS. The candidate genes *LINC01550* and *BCL11B* contained multiple CpG sites. Columns and rows represent one CpG loci of the listed candidate gene.

**Additional file 10: Fig. S6.** Expression correlation between *BCL11B* & *C14ORF64* (*LINC01550*). Expression correlation between *BCL11B* & *C14ORF64* (*LINC01550*) in 426 human datasets with 42563 samples from R2: Genomics analysis and visualization platform (https://hgserver1.amc.nl/cgi-bin/r2/main.cgi).

**Additional file 11:** Material and Methods.

## Data Availability

The data are available from the Gene Expression Omnibus (TBD) in a MIAME compliant format.

## Abbreviations

DMPs: Differentially methylated positions
DMRs: Differentially methylated regions
LINC: Long intergenic non-protein coding
PC: Principal component
PCA: Principal component analysis
STAT3: Signal transducer and activator of transcription 3
TCR: T-cell receptor
T-LGLL: T-cell large granular lymphocytic leukemia
TSS: Transcription start site
