# Publisher Correction: Epigenome-wide analysis of T-cell large granular lymphocytic leukemia identifies BCL11B as a potential biomarker

**DOI:** 10.1186/s13148-022-01405-5

**Published:** 2023-01-06

**Authors:** Patricia Johansson, Teresa Laguna, Julio Ossowski, Vera Pancaldi, Martina Brauser, Ulrich Dührsen, Lara Keuneke, Ana Queiros, Julia Richter, José I. Martín-Subero, Reiner Siebert, Brigitte Schlegelberger, Ralf Küppers, Jan Dürig, Eva M. Murga Penas, Enrique Carrillo-de Santa Pau, Anke K. Bergmann

**Affiliations:** 1 Faculty of Medicine, Institute of Cell Biology (Cancer Research), University of Duisburg-Essen, Virchowstr. 177, 45122 Essen, Germany; 2 Computational Biology Group, Precision Nutrition and Cancer Research Program, IMDEA Food Institute, 28049 Madrid, Spain; 3 Institute for Human Genetics, Christian-Albrechts-University Kiel and University Hospital Schleswig Holstein, Campus Kiel, Kiel, Germany; 4 Institute of Human Genetics, Medical School Hannover (MHH), Hannover, Germany; 5 Centre de Recherches en Cancérologie de Toulouse (CRCT), Université de Toulouse, CNRS, Université Toulouse III-Paul Sabatier, Centre de Recherches en Cancérologie de Toulouse, INSERM U1037, 31037 Toulouse, France; 6 Barcelona Supercomputing Center, 08034 Barcelona, Spain; 7 Department of Hematology, University Hospital Essen, University of Duisburg-Essen, Essen, Germany; 8 Institute for Pathology, Christian-Albrechts-University Kiel and University Hospital Schleswig Holstein, Campus Kiel, Kiel, Germany; 9 Institut d’Investigacions Biomediques August Pi I Sunyer (IDIBAPS), University of Barcelona, 08036 Barcelona, Spain; 10 Institució Catalana de Recerca i Estudis Avançats (ICREA), 08010 Barcelona, Spain; 11 Department of Internal Medicine, University Hospital Essen, St. Josef-Krankenhaus, University Medicine Essen, Essen, Germany; 12 Present Address: Institute of Human Genetics, University of Ulm and University Medical Center Ulm, Ulm, Germany

**Correction to: Clinical Epigenetics (2022) 14:148** 10.1186/s13148-022-01362-z

Following publication of the original article [1], the author noticed an error in the figure. In the published version, the image of Fig. 5 has been repeated twice. The author has informed to remove the duplicated image during correction process. The typesetter has inadvertently missed to correct the figure. However, the text citation and caption of the figure seem to be correct. The corrected Fig. 5 has been published with this erratum.

**Fig. 5:**
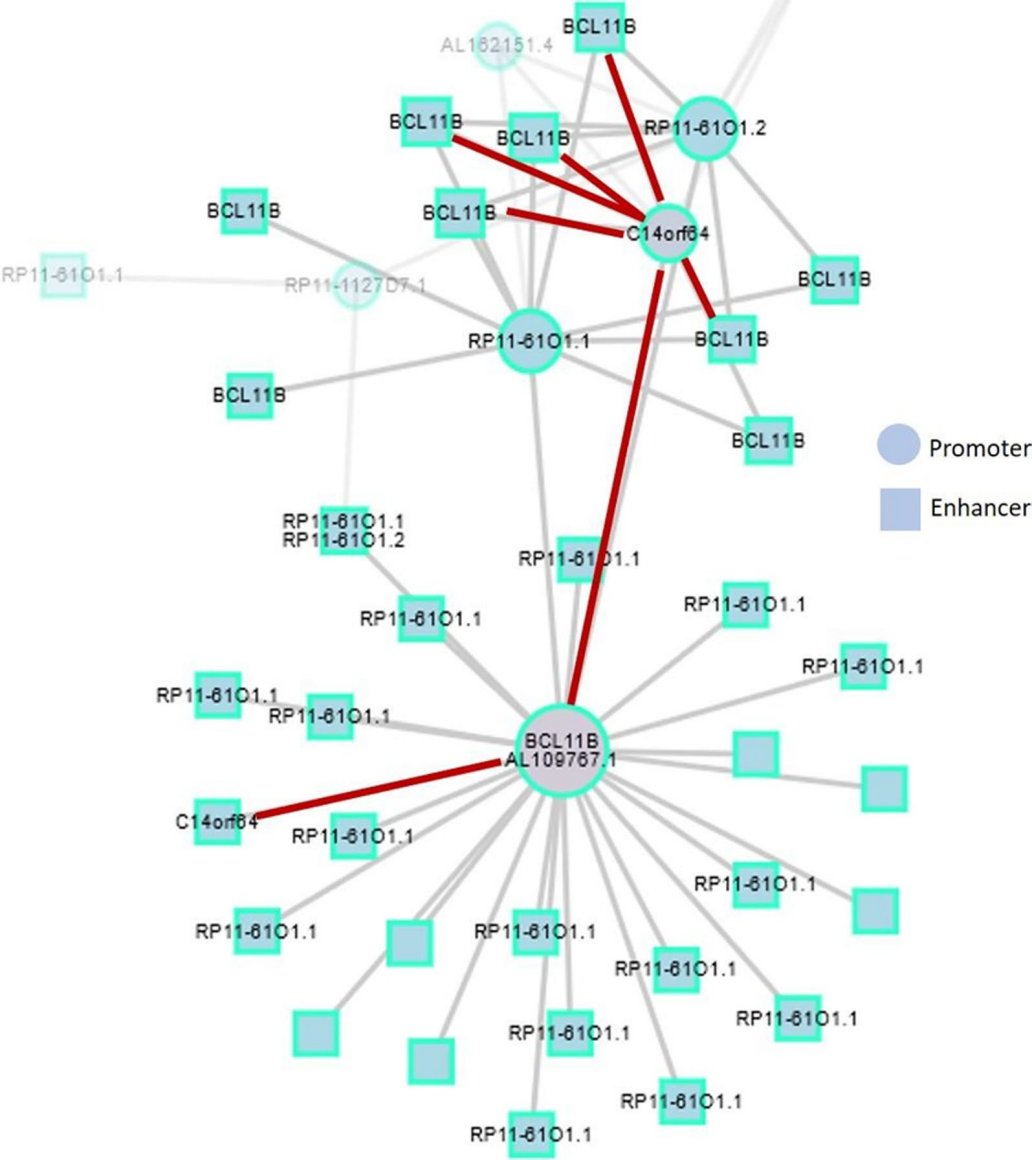
Interaction of the gene BCL11B with other genes in CD8^+^ cells. BCL11B long-range interactions from Promoter Capture Hi-C for total CD8-positive T cells (red: BCL11B–C14orf64 (LINC01550) interactions). Genomic regions are depicted in blue circles (promoter region) or blue square (enhancer region)

The original has been corrected.
