# Supplementary figures and images for: Epigenome-wide analysis of T-cell large granular lymphocytic leukemia identifies BCL11B as a potential biomarker
Source: Clin Epigenetics. 2022 Nov 14;14:148. doi: 10.1186/s13148-022-01362-z (PMC9664638; doi:10.1186/s13148-022-01362-z)

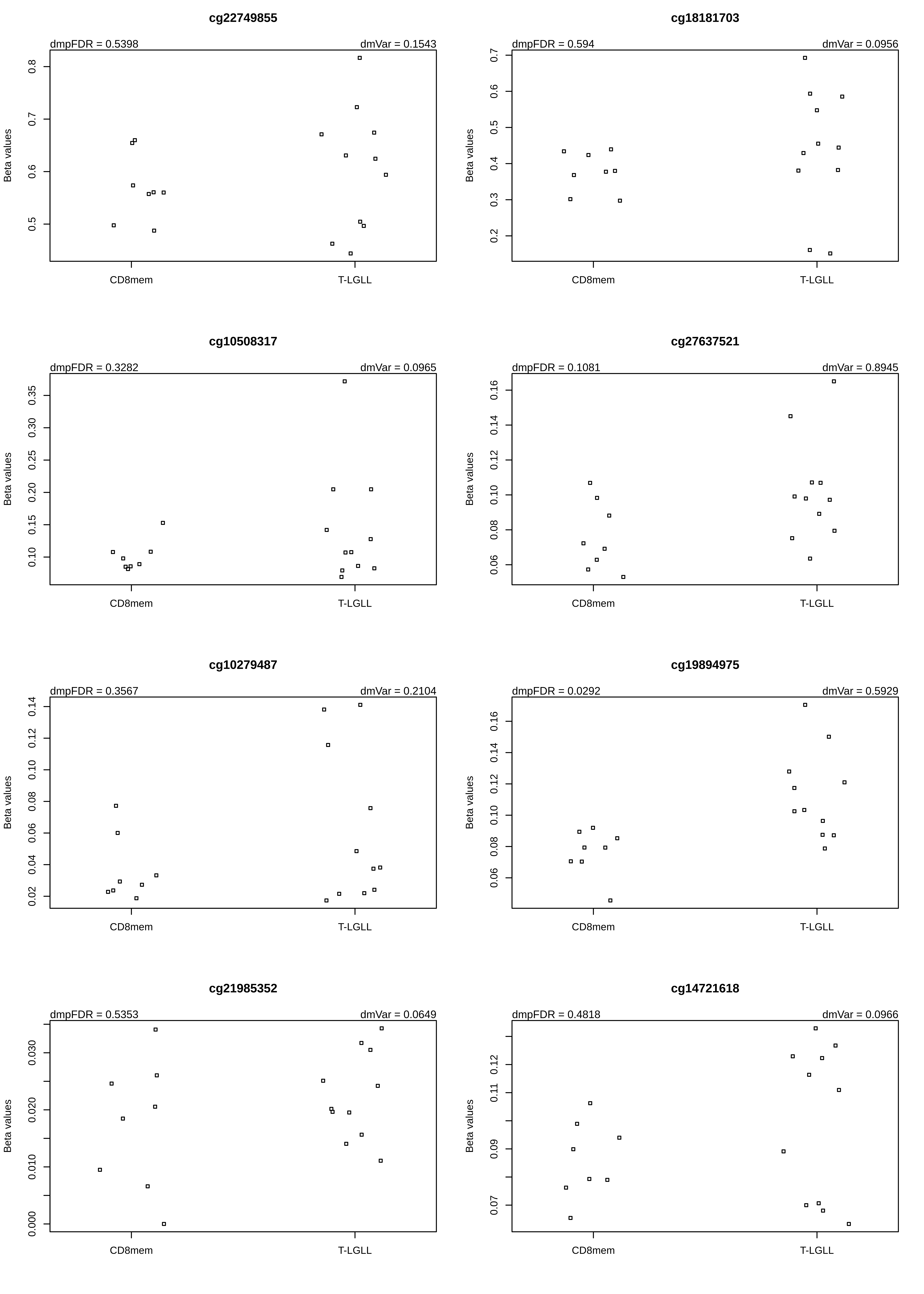

Supplement: Supplementary file 2 — Additional file 2: Fig. S2. Differential methylation of CpG loci in the SOCS3 promoter in T-LGL samples. Comparison of CpG methylation (beta-value) for CpGs in the SOCS3 promoter between CD8+ memory cells (CD8mem) and T-LGLL samples (LGL). On top, adjusted p val of differential methylation analysis (dmpFDR, top left) and adjusted p value of differential variability analysis (dmVar, top right). [file 13148_2022_1362_MOESM2_ESM.tif]

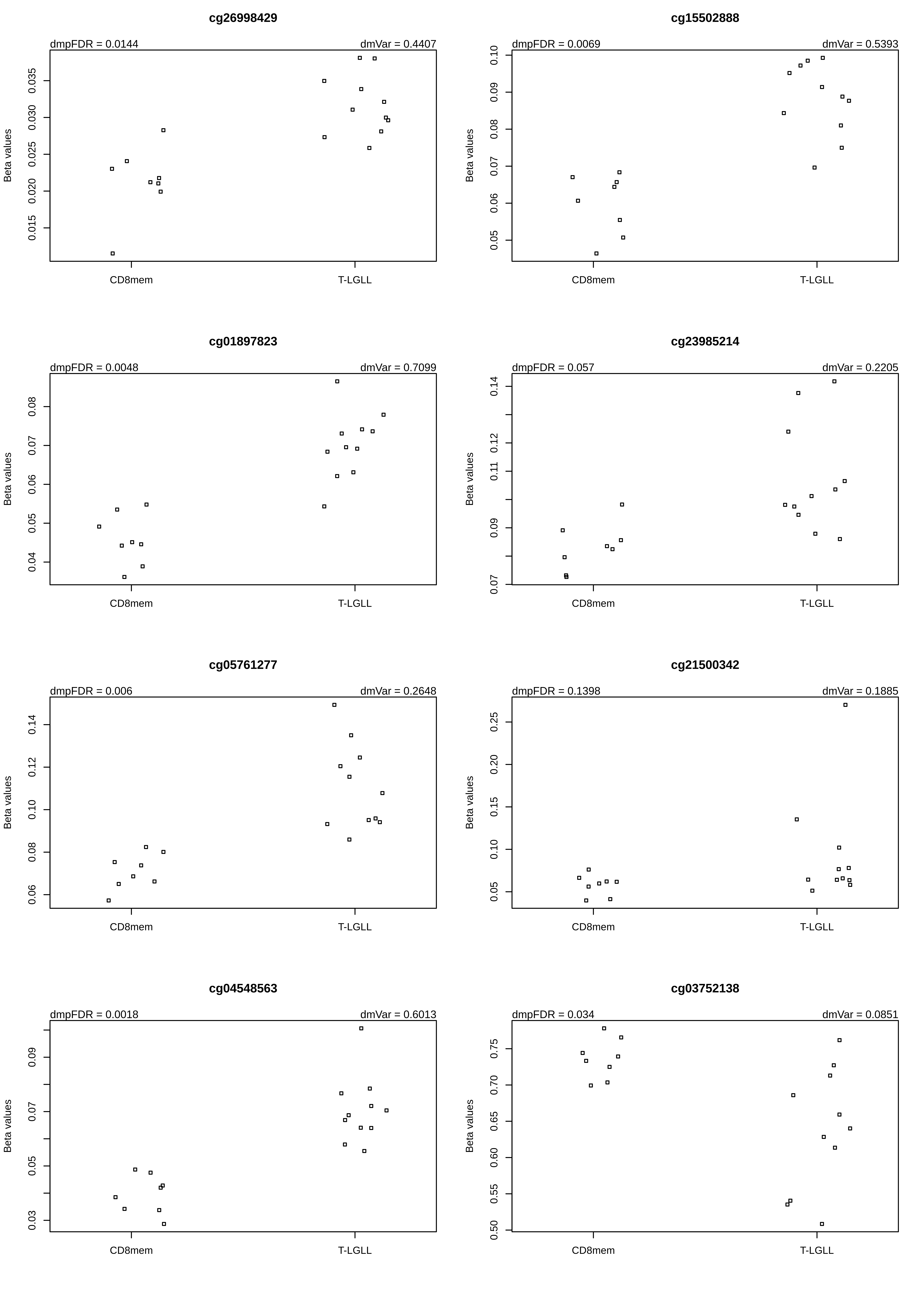

Supplement: Supplementary file 3 — Additional file 3: Fig. S2. Differential methylation of CpG loci in the SOCS3 promoter in T-LGL samples. Comparison of CpG methylation (beta-value) for CpGs in the SOCS3 promoter between CD8+ memory cells (CD8mem) and T-LGLL samples (LGL). On top, adjusted p val of differential methylation analysis (dmpFDR, top left) and adjusted p value of differential variability analysis (dmVar, top right). [file 13148_2022_1362_MOESM3_ESM.tif]

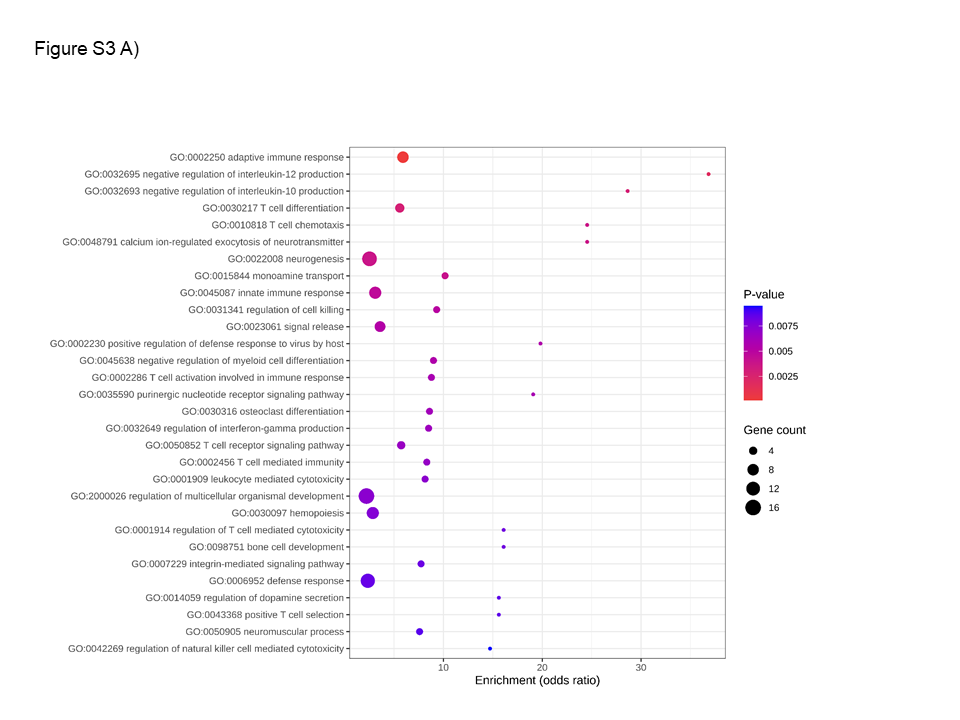

Supplement: Supplementary file 4 — Additional file 4: Fig. S3A. Gene Ontology analysis of genes hyper- (A) and hypomethylated (B) of T-LGL patients. Significant Biological processes (GO database) enriched in genes associated with significantly differentially methylated CpG loci in T-LGL. Enrichment represented as odds ratio. Point size represents the gene count of each pathway. Enrichment p value obtained by overrepresentation analysis [30], represented by point color. A Gene Ontology analysis of hypermethylated genes in T-LGL. [file 13148_2022_1362_MOESM4_ESM.tif]

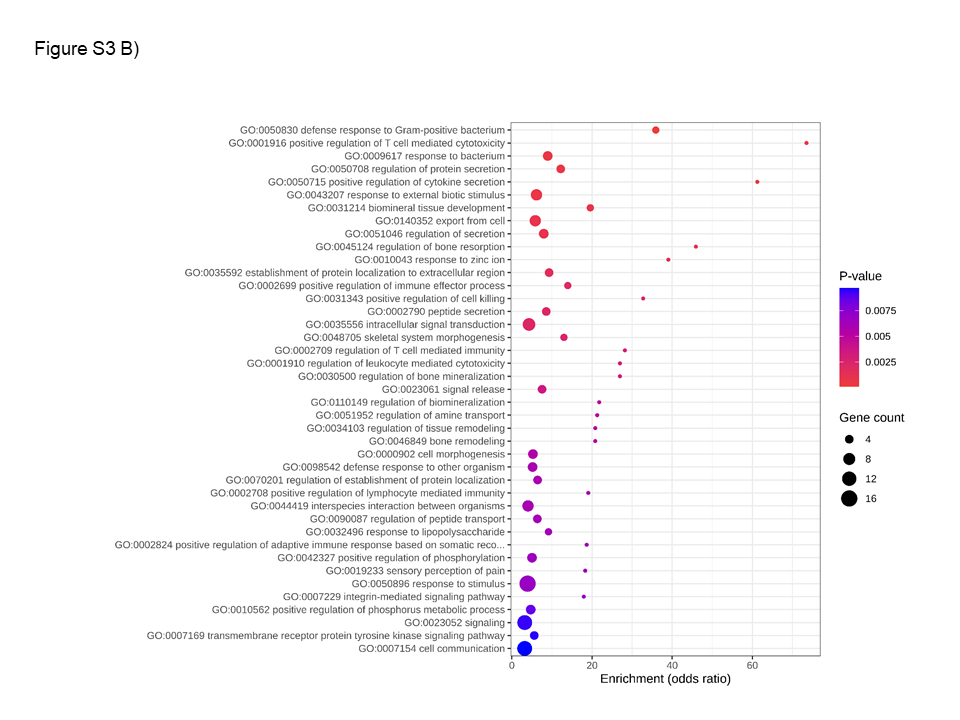

Supplement: Supplementary file 5 — Additional file 5: Fig. S3B. Gene Ontology analysis of genes hyper- (A) and hypomethylated (B) of T-LGL patients. Significant Biological processes (GO database) enriched in genes associated with significantly differentially methylated CpG loci in T-LGL. Enrichment represented as odds ratio. Point size represents the gene count of each pathway. Enrichment p value obtained by overrepresentation analysis [30], represented by point color. B Gene Ontology analysis of hypomethylated genes in T-LGL. [file 13148_2022_1362_MOESM5_ESM.tif]

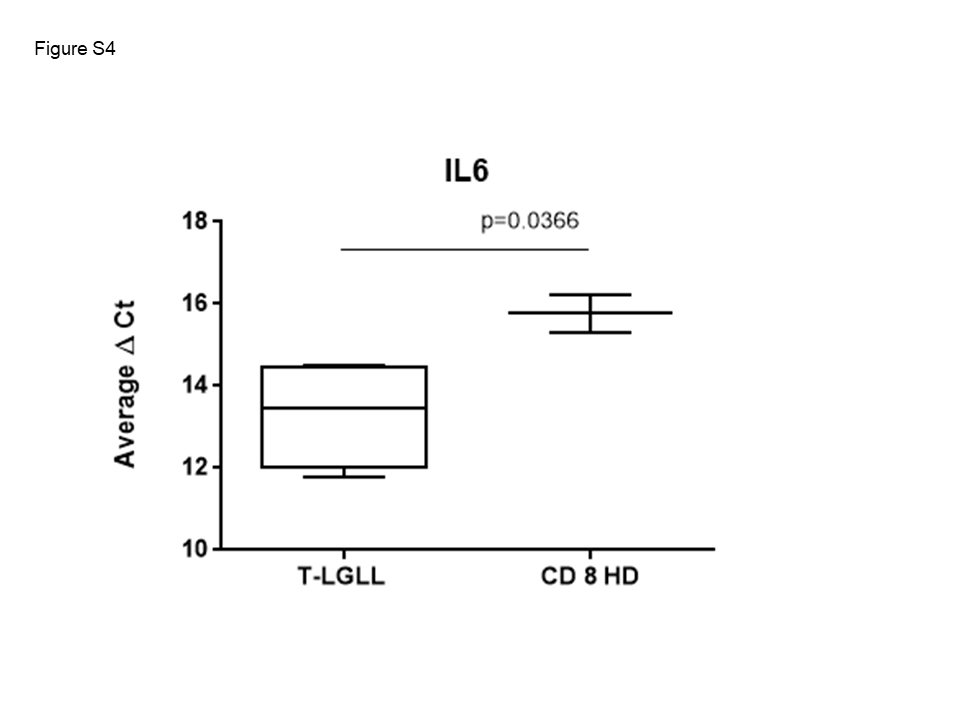

Supplement: Supplementary file 6 — Additional file 6: Fig. S4. Differential gene expression of IL6 between T-LGL and healthy donor-derived CD8+ memory T cells. Differential gene expression for IL6 was measured by qPCR. Bulk CD8+ cells from two healthy donors were used for comparison. In line with previous publications, the T-LGLL cohort analyzed exhibits a higher IL6 expression compared to healthy donor-derived C8+ cells. HD Healthy donor. [file 13148_2022_1362_MOESM6_ESM.tif]

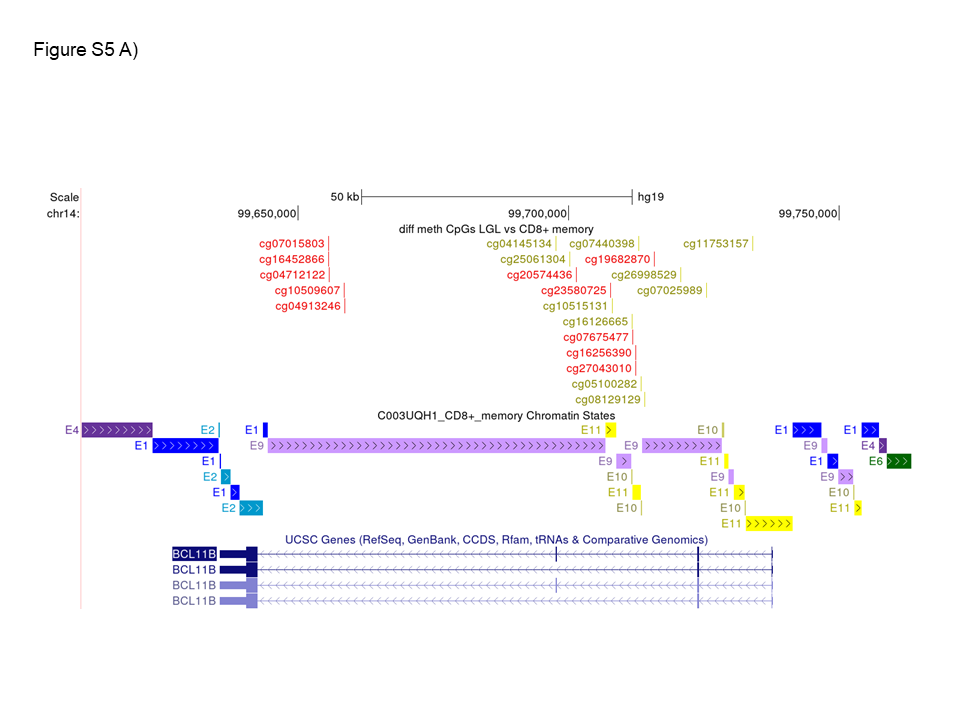

Supplement: Supplementary file 7 — Additional file 7: Fig. S5A. (A and B): Location of differentially methylated CpG loci in the genes BCL11B and C14orf64 (LINC01550). A Significant differentially methylated CpGs in BCL11B (T-LGLL compared to CD8+. memory T cells) were located in the gene body and assigned as enhancers by ENCODE, which match as enhancers in CD8-positive memory cells. [file 13148_2022_1362_MOESM7_ESM.tif]

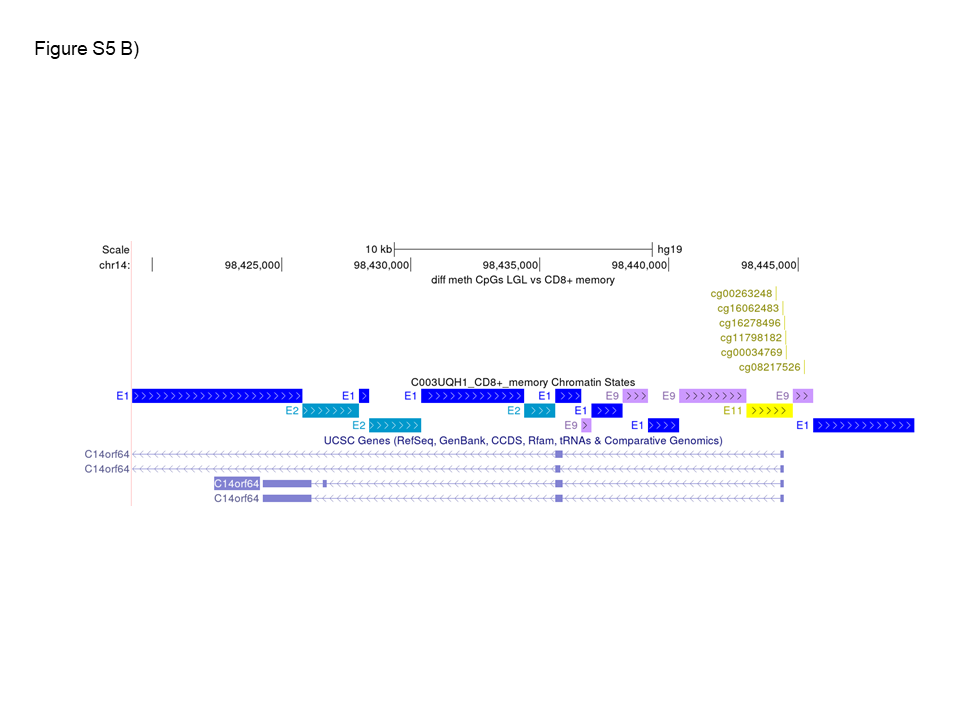

Supplement: Supplementary file 8 — Additional file 8: Fig. S5B. (A and B): Location of differentially methylated CpG loci in the genes BCL11B and C14orf64 (LINC01550). B Significant differentially methylated CpGs in C14orf64 (LINC01550) (T-LGL compared to CD8 pos. memory T cells). [file 13148_2022_1362_MOESM8_ESM.tif]

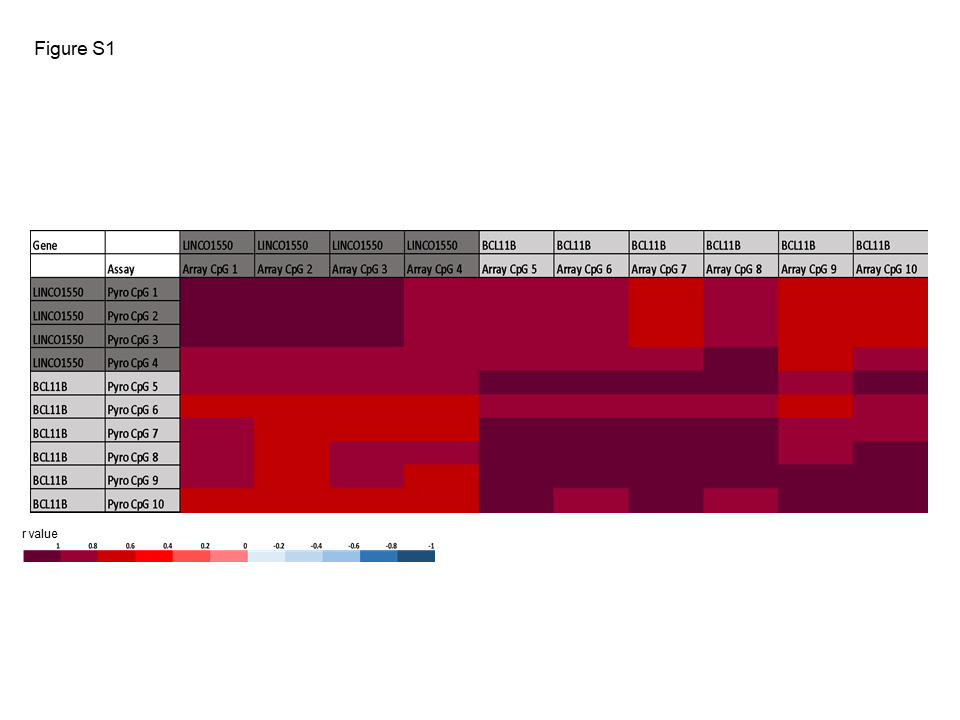

Supplement: Supplementary file 9 — Additional file 9: Fig. S1. Correlation of bisulfite Pyrosequencing (BPS) and methylation Array DNA methylation levels. The correlation matrix shows the Pearson correlation coefficient (r: 1 (red) to − 1 (blue) among all CpG loci analyzed by BPS. The candidate genes LINC01550 and BCL11B contained multiple CpG sites. Columns and rows represent one CpG loci of the listed candidate gene. [file 13148_2022_1362_MOESM9_ESM.tif]

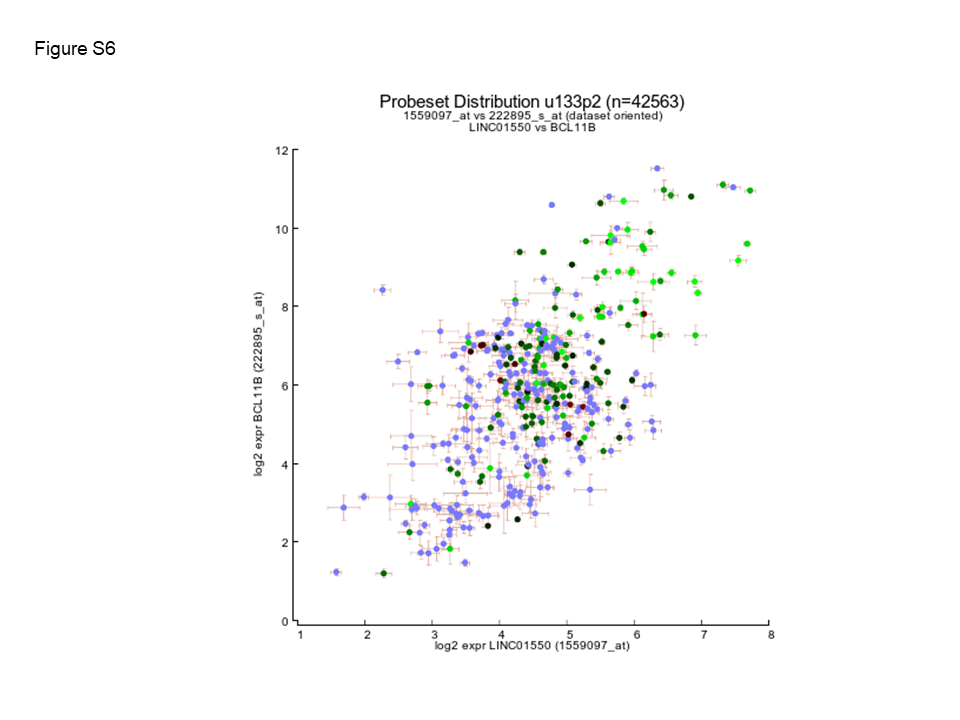

Supplement: Supplementary file 10 — Additional file 10: Fig. S6. Expression correlation between BCL11B & C14ORF64 (LINC01550). Expression correlation between BCL11B & C14ORF64 (LINC01550) in 426 human datasets with 42563 samples from R2: Genomics analysis and visualization platform (https://hgserver1.amc.nl/cgi-bin/r2/main.cgi). [file 13148_2022_1362_MOESM10_ESM.tif]
