# Supplementary material for: Epigenome-wide analysis of T-cell large granular lymphocytic leukemia identifies BCL11B as a potential biomarker
Source: Clin Epigenetics. 2022 Nov 14;14:148. doi: 10.1186/s13148-022-01362-z (PMC9664638; doi:10.1186/s13148-022-01362-z)
Supplement: Supplementary file 11 — Additional file 11: Material and Methods. [file 13148_2022_1362_MOESM11_ESM.docx]

**Supplemental file**

**Material and Methods**

Statistical analyses of the HumanMethylation450 Bead Chip DNA methylation data

Raw files (.idat) were processed with R software version ≥ 4.0 [1] using R packages minfi, limma, missMethyl, DMRcate, reshape2 and ddplyr [2-7]. Intensities of methylated and unmethylated signals were obtained after background correction. Data were normalized using the “illumina” method implemented in *minfi*, which performs background subtraction and control normalization. Positions with detection p-values > 0.01, in sexual chromosomes, matching SNPs with MAF >0 and multimappers were then filtered out, leaving a total of 401,778 CpGs for further analysis. A principal component analysis (PCA) was calculated using the beta-values of the filtered probes with a sd_i_/sd_max_ > 0.4, using *prcomp* from the R base package *stats*, without centering or scaling the values. A differential methylation analysis was performed a) individually by CpGs using the M-values by means of the linear model with posterior Bayesian variance fitting implemented in *limma* and b) by region using the *dmrcate* package, which consists in smoothing the moderated t-test values per CpG from the limma method, comparing with an unweighted null distribution, calculating significance via a Satterthwaite approximation (Satterthwaite, 1946) and merging significant CpGs in regions where the maximum distance between CpG sites was determined by the parameter lambda=1000. In addition to lambda, parameter C=2 for *dmrcate* was left as default as suggested by running *cpg.annotate* from the same package.

*Both packages include in their analysis a multiple testing correction of p-values using the Benjamini-Hochberg (BH) method. Only corrected p-values (adj p-val < 0.005) were considered for significance*.

For the individual CpG analysis, we obtained from the Illumina 450k array annotation (IlluminaHumanMethylation450kanno.ilmn12.hg19 R annotation package) the genes overlapping to the 2,216 DMPs. We performed a ranking of the genes according to the proportion of the significant DMPs from the total analyzed for each gene (from the filtered 401,778 probes), and into for the hypermethylated or hypomethylated genes depending on the ratio of hyper- or hypomethylated CpGs in T-LGL samples for each gene. We considered significant hyper- or hypomethylated genes when contained > 1 significant CpG or with a ratio of significant CpGs in promoters > 0.33 (at least ⅓ of the promoter CpGs were significant).

All plots were generated in R by means of the packages *ggplot2*, *ComplexHeatmap*, *circlize*, *threejs, htmlwidgets*, *RColorBrewer* [8–13].

Functional enrichment analysis

Different methods were employed to perform functional enrichment analyses of the differentially methylated CpG sites. A method specific for differential methylation implemented in *missMethyl* was used in an individual *dmp* (‘differentially methylated probes’) manner and using the differentially methylated regions (*gometh* and *goregion* functions, respectively). The most specific results were obtained by selecting the genes containing the *dmp* and classifying them according to their proportion of significant positions in promoters or enhancers or gene body. We selected those genes for enrichment analysis with proportion >0.3 CpGs significant in promoters (*TSS200* and *5’UTR* features in the *UCSC_RefGene_group* category in *IlluminaHumanMethylation450kanno.ilmn12.hg19* R annotation package)*,* or with a proportion of significant CpGs in enhancers >0.4 (*Enhancer* = TRUE in the annotation package). Then, genes were classified as hypermethylated or hypomethylated in T-LGLL samples compared to non-neoplastic samples. Overrepresentation analysis was carried out by means of the *GOstats* package [14], using as gene *universe* the genes assigned to each of the 401,778 probes, using the *conditional* mode, where child pathways are considered independent of their corresponding parent pathways.

Pyrosequencing

Pyrosequencing was used to validate data obtained from the methylation array analyses. The validation cohort was extended by including six additional patients; sequential samples were available for two of these patients. Samples were bisulfite converted using the EpiTectPlus Bisulfite Conversion Kit (Qiagen) according to manufacturer’s instructions. PCR amplification with biotinylated primers (Supplemental Table S4), pyrosequencing, and data analysis were performed with the PyroMark Q96 sequencer (Qiagen) as previously described [15, 16]. Shortly, the locus-specific assays for each of the selected loci were designed with the PyroMark assay design software (Qiagen, version 2.0). Primers without SNPs and low penalty overall quality parameters were ordered from Biomers (Ulm, Deutschland). The PCR conditions for each primer set were optimized using the PyroMark PCR Kit (Qiagen). Optimized primers were tested by gel electrophoresis. Validation of ten differentially methylated CpGs in the genes *BCL11B* and *LINC01550* was performed by bisulfite pyrosequencing (BPS) (Supplemental Tables S4 and S5).

Statistical analyses of pyrosequencing data

The PyroMark Q96 ID software was used to quantify DNA methylation levels. The Pearson correlation coefficient was applied for correlation of the BPS values with the values obtained in the methylation array (Supplemental Figure S1 and Supplemental Table S6). Statistical analyses were performed using SPSS 26.0 (IBM SPSS, Chicago, IL, USA).

Analysis of differential gene expression by reverse transcription real-time PCR (qPCR)

Differential gene expression between sorted T-LGLL cells of eleven patients and four mature T cell subsets from five healthy donors and bulk CD8 T cells from two additional donors was analyzed for 9 genes using reverse transcription real-time PCR. Total RNA was extracted using peqGOLD Micro Spin Columns (Peqlab, Erlangen, Germany) following manufacturer’s instructions including on-column DNase digestion (Qiagen). Synthesis of cDNA was performed with Sensiscript (Qiagen) using anchored oligo(dT) primer (Thermo Scientific, Waltham, MA, USA). Target gene expression was analyzed in triplicates using TaqMan Universal PCR Master Mix No AmpErase UNG (Applied Biosystems - Thermo Scientific). GAPDH was used as housekeeping gene. Target gene detection was performed with TaqMan assays (Supplemental Table S8) (Applied Biosystems – Thermo Scientific). 96-well plates were run on an ABI PRISM 7900HT system (Applied Biosystems) and cycle threshold (Ct) values were calculated in SDS software (v2.2, Applied Biosystems - Thermo Scientific).

Analysis of differential gene expression by reverse transcription real-time PCR (qPCR)

Differential gene expression between sorted T-LGLL cells of eleven patients and four mature T cell subsets from five healthy donors and bulk CD8 T cells from two additional donors was analyzed for 9 genes using reverse transcription real-time PCR. Total RNA was extracted using peqGOLD Micro Spin Columns (Peqlab, Erlangen, Germany) following manufacturer’s instructions including on-column DNase digestion (Qiagen). Synthesis of cDNA was performed with Sensiscript (Qiagen) using anchored oligo(dT) primer (Thermo Scientific, Waltham, MA, USA). Target gene expression was analyzed in triplicates using TaqMan Universal PCR Master Mix No AmpErase UNG (Applied Biosystems - Thermo Scientific). GAPDH was used as housekeeping gene. Target gene detection was performed with TaqMan assays (Supplemental Table S8) (Applied Biosystems – Thermo Scientific). 96-well plates were run on an ABI PRISM 7900HT system (Applied Biosystems) and cycle threshold (Ct) values were calculated in SDS software (v2.2, Applied Biosystems - Thermo Scientific).

1. R Core Team. R: A language and environment for statistical computing. R Foundation for Statistical Computing, Vienna, Austria. 2021.

2. Aryee MJ, Jaffe AE, Corrada-Bravo H, Ladd-Acosta C, Feinberg AP, Hansen KD, Irizarry RA. Minfi: a flexible and comprehensive Bioconductor package for the analysis of Infinium DNA methylation microarrays. Bioinformatics. 2014;30:1363–9. doi:10.1093/bioinformatics/btu049.

3. Ritchie ME, Phipson B, Di Wu, Hu Y, Law CW, Shi W, Smyth GK. limma powers differential expression analyses for RNA-sequencing and microarray studies. Nucleic Acids Res. 2015;43:e47. doi:10.1093/nar/gkv007.

4. Phipson B, Maksimovic J, Oshlack A. missMethyl: an R package for analyzing data from Illumina’s HumanMethylation450 platform. Bioinformatics. 2016;32:286–8. doi:10.1093/bioinformatics/btv560.

5. Peters TJ, Buckley MJ, Statham AL, Pidsley R, Samaras K, V Lord R, et al. De novo identification of differentially methylated regions in the human genome. Epigenetics Chromatin. 2015;8:6. doi:10.1186/1756-8935-8-6.

6. Wickham H. Reshaping Data with the reshape Package. Journal of Statistical Software. 2007:1–20.

7. Hadley Wickham, Romain François, Lionel Henry and Kirill Müller. dplyr: A Grammar of Data Manipulation. R package version 1.0.7. [https://CRAN.R-project.org/package=dplyr](https://cran.r-project.org/package=dplyr). 2021.

8.   Wickham H. ggplot2: Elegant graphics for data analysis. Cham: Springer International Publishing; 2016.

9.   Gu Z, Eils R, Schlesner M. Complex heatmaps reveal patterns and correlations in multidimensional genomic data. Bioinformatics. 2016;32:2847–9. doi:10.1093/bioinformatics/btw313.

10.  Gu Z, Gu L, Eils R, Schlesner M, Brors B. circlize Implements and enhances circular

visualization in R. Bioinformatics. 2014;30:2811–2. doi:10.1093/bioinformatics/btu393.

11.  Neuwirth E. RColorBrewer: <https://rdrr.io/cran/RColorBrewer/man/ColorBrewer.html;2014>.

12. Vaidyanathan. <https://www.htmlwidgets.org/showcase_leaflet.html;2020>.

13.  B. W. Lewis. Package ‘threejs’. <https://bwlewis.github.io/rthreejs/>. 2020.

14. Falcon S, Gentleman R. Using GOstats to test gene lists for GO term association.

Bioinformatics. 2007;23:257–8. doi:10.1093/bioinformatics/btl567.

15. Quantitative comparison of DNA methylation assays for biomarker development and

clinical applications. Nat Biotechnol. 2016;34:726–37. doi:10.1038/nbt.3605.

16. Tost J, Gut IG. DNA methylation analysis by pyrosequencing. Nat Protoc. 2007;2:2265–75. doi:10.1038/nprot.2007.314.

Supplementary Figure legends:

Figure S1: **Correlation of bisulfite Pyrosequencing (BPS) and methylation Array DNA methylation levels.**

The correlation matrix shows the Pearson correlation coefficient (r: 1 (red) to -1 (blue) among all CpG loci analyzed by BPS. The candidate genes *LINC01550* and *BCL11B* contained multiple CpG sites. Columns and rows represent one CpG loci of the listed candidate gene.

Figure S2: **Differential methylation of** **CpG loci in the *SOCS3* promoter in T-LGL samples.**

Comparison of CpG methylation (beta-value) for CpGs in the SOCS3 promoter between CD8^+^ memory cells (*CD8mem*) and T-LGLL samples (*LGL*). On top, adjusted p-val of differential methylation analysis (*dmpFDR*, top left) and adjusted p-value of differential variability analysis (*dmVar*, top right).

Figure S3: **Gene Ontology analysis of genes hyper- (A) and hypomethylated (B) of T-LGL patients.**

Significant Biological processes (GO database) enriched in genes associated with significantly differentially methylated CpG loci in T-LGL. Enrichment represented as odds ratio. Point size represents the gene count of each pathway. Enrichment p-value obtained by overrepresentation analysis [30], represented by point color. A) Gene Ontology analysis of hypermethylated genes in T-LGL; B) Gene Ontology analysis of hypomethylated genes in T-LGL.

Figure S4: **Differential gene expression of *IL6* between T-LGL and healthy donor derived CD8^+^ memory T cells**

Differential gene expression for *IL6* was measured by qPCR. Bulk CD8^+^ cells from two healthy donors were used for comparison. In line with previous publications, the T-LGLL cohort analyzed exhibits a higher *IL6* expression compared to healthy donor derived C8+ cells.

Abrev.: HD - healthy donor

Figure S5 (A and B): **Location of differentially methylated CpG loci in the genes *BCL11B* and *C14orf64 (LINC01550)***

(A) Significant differentially methylated CpGs in *BCL11B* (T-LGLL compared to CD8+. memory T cells) were located in the gene body and  assigned as enhancers by ENCODE, which match as enhancers in CD8-positive memory cells. (B) Significant differentially methylated CpGs in *C14orf64 (LINC01550)* (T-LGL compared to CD8 pos. memory T cells)

Figure S6: **Expression correlation between BCL11B & C14ORF64 (LINC01550)**

Expression correlation between BCL11B & C14ORF64 (LINC01550) in 426 human datasets with 42563 samples from *R2: Genomics analysis and visualization platform* (<https://hgserver1.amc.nl/cgi-bin/r2/main.cgi>).
